# Models of Workplace Incivility: The Relationships to Instigated Incivility and Negative Outcomes

**DOI:** 10.1155/2015/920239

**Published:** 2015-10-18

**Authors:** Kristoffer Holm, Eva Torkelson, Martin Bäckström

**Affiliations:** Department of Psychology, Lund University, P.O. Box 213, 221 00 Lund, Sweden

## Abstract

The aim of the study was to investigate workplace incivility as a social process, examining its components and relationships to both instigated incivility and negative outcomes in the form of well-being, job satisfaction, turnover intentions, and sleeping problems. The different components of incivility that were examined were experienced and witnessed incivility from coworkers as well as supervisors. In addition, the organizational factors, social support, control, and job demands, were included in the models. A total of 2871 (2058 women and 813 men) employees who were connected to the Swedish Hotel and Restaurant Workers Union completed an online questionnaire. Overall, the results from structural equation modelling indicate that whereas instigated incivility to a large extent was explained by witnessing coworker incivility, negative outcomes were to a high degree explained by experienced supervisor incivility via mediation through perceived low social support, low control, and high job demands. Unexpectedly, the relationships between incivility (experienced coworker and supervisor incivility, as well as witnessed supervisor incivility) and instigated incivility were moderated by perceived high control and high social support. The results highlight the importance of including different components of workplace incivility and organizational factors in future studies of the area.

## 1. Introduction

The aim of the present study was to explore workplace incivility as a social process, including experienced as well as witnessed incivility from coworkers and supervisors and its relationships to instigated incivility and negative outcomes in the form of well-being, job satisfaction, turnover intentions, and sleeping problems. The goal was to create comprehensive models including direct relationships between workplace incivility and its outcomes, as well as mediation and moderation of organizational factors. This adds to the current literature through including different components of workplace incivility as well as organizational factors in the same models to explain instigated incivility and negative outcomes. Workplace incivility has been defined as “… low-intensity deviant behavior with ambiguous intent to harm the target, in violation of workplace norms for mutual respect. Uncivil behaviors are characteristically rude and discourteous, displaying a lack of regard for others” [1]. Incivility, as a covert form of aggression, demarcates from other forms of overt workplace aggression in that it can be ambiguous and of lower intensity and does not necessarily need to be intended to harm [2]. Despite this, incivility has been equated to the severity of workplace bullying on the outcomes of job satisfaction and of turnover intentions [3]. Examples of such covert behaviours are rude looks or ignoring someone, compared to overt behaviors like yelling [3].

Beyond experienced incivility, research has also been requested on perspectives focusing on the bystanders and perpetrators as well as the organizational context as components of the incivility process [4].

Studies have approached the social process of incivility, exploring it as a group-level phenomenon [1, 5]. Andersson and Pearson [1] raise the issue of how incivility may manifest in the form of a reciprocal social process between involved individuals. The authors theorized about a negative spiral, where incivility can create escalating responses of growing workplace aggression nourishing interpersonal conflicts. Further research has since supported this notion, indicating that the destructive spiral of workplace incivility, may be a building block in a negative work environment [6]. Being targeted by incivility has been shown to lead to negative emotions that subsequently relate to aggression [7].

When investigating the escalation of workplace aggression, Taylor and Kluemper [8] reported findings additionally supporting the relationship between perceived incivility and workplace aggression, when incivility is seen as a mediator between role stress and aggression. A stressful environment would thus induce higher ratings of instigated incivility, leading to further reciprocal behaviours, resulting in increased aggression. Some scholars have viewed incivility as a stressor (e.g., [5, 9]). In relation to stress and strain research Karasek and Theorell's [10] demand-control-support (DCS) model has been often applied in the literature of occupational health psychology. Earlier research [11] on the onset of bullying, as an overt form of aggression, included variables from the DCS model. Thus, it is interesting to include organizational factors from the model in the investigation of a covert form of aggression such as workplace incivility.

Considering Andersson and Pearson's [1] reasoning about an uncivil spiral with “tit for tat” responses, the self-sustaining nature of such a spiral highlights the risk of instigated incivility as an outcome, related to either experienced or witnessed incivility in the workplace. In an interview-based study by Pearson et al. [2], it was found that witnesses to incivility modelled their behaviour after their observations, retaliating uncivil acts. In line with this, Ferguson and Barry [12] reported that individuals in highly cohesive groups were more likely to adopt uncivil behaviour if witnessing it. In the present study we investigated if experienced and witnessed incivility is related to instigated incivility and negative outcomes in models including four organizational factors, social support from coworkers, social support from superiors, control, and job demands. In the study, we tested both direct relations of experienced and witnessed incivility, as well as mediation and moderation of organizational variables towards the outcomes. Thus, the first hypothesis tested was:

 experienced and witnessed workplace incivility, from coworker or supervisor, is directly related to instigated incivility.

In the field of workplace incivility, more research on possible mediators has been requested [13]. Negative emotions, however, have been shown to mediate the relationship between coworker incivility and increased deviant behaviour [14]. Schilpzand et al. [15] argue that most studies have not investigated the mediating mechanisms for why certain antecedent constructs would lead to incivility. In addition, not much work has been conducted on organizational factors such as job demands, control, and social support as mediators of workplace incivility. Testing possible mediation of these factors would be an addition to the field. Thus, the second hypothesis was:

 organizational factors (social support from coworker, social support from supervisor, control, and job demands) mediate the relationships between experienced and witnessed workplace incivility (from coworker or supervisor) and instigated incivility.

Control has previously been shown to buffer effects of job demands on being targeted by bullying in the workplace [11], and psychosocial factors have also been approached as moderators in the relationship between incivility and instigated counterproductive work behaviour [14]. In line with this, the third hypothesis was:

 organizational factors (social support from coworker, social support from supervisor, control, and job demands) moderate the relationships between experienced and witnessed workplace incivility (from coworker or supervisor) and instigated incivility.

A high level of incivility has been linked to a number of negative outcomes. In the present study, we focus on negative outcomes in the form of low well-being, low job satisfaction, turnover intentions, and sleeping problems. Incivility is negatively related to both mental and physical well-being [9, 16]. Studies also consistently report that individuals subjected to workplace incivility, from both a target and an instigator perspective, experience lower job satisfaction [16, 17]. Lim et al. [9] found that incivility impact the entire organization in form of lower levels of job satisfaction and mental health, even when controlling for job stress. The relationship between job satisfaction and witnessed incivility has since been supported [18].

Being the victim of uncivil behaviour has been related directly to turnover intentions [6, 19] and incivility from a supervisor has shown to be stronger related to turnover intentions than coworker incivility [20].

Moreover, having troubles with sleep has previously been shown to be strongly related to other types of workplace aggression, such as bullying [21, 22]. Similarly, both experienced and witnessed bullying has been studied, where witnessing bullying relates to detrimental outcomes [23, 24]. As follows to this, the fourth hypothesis was:

 experienced and witnessed workplace incivility, from coworker or supervisor, is directly related to employees' negative outcomes (well-being, job satisfaction, and turnover intentions, as well as sleeping problems).

Emotional and organizational support has previously been found to mediate the effects between experienced workplace incivility and negative outcomes [25]. Social and organizational support has also been approached as both a mediator and a moderator in the research on workplace bullying and negative outcomes [23, 26]. The DCS model, concerning the variables of support, control and job demands, is well established in the workplace literature and has previously been tied to well-being [27]. In light of this, it serves important to include these variables in the present study. Thus, the fifth hypothesis was:

 organizational factors (social support from coworker, social support from supervisor, control, and job demands) mediate the relationships between experienced and witnessed workplace incivility (from coworker or supervisor) and negative outcomes (well-being, job satisfaction, turnover intentions, and sleeping problems).

Additionally, we tested a sixth hypothesis:

 organizational factors (social support from coworker, social support from supervisor, control, and job demands) moderate the relationships between experienced and witnessed workplace incivility (from coworker or supervisor) and negative outcomes (well-being, job satisfaction, turnover intentions, and sleeping problems).

The population of the present study consisted of individuals employed in the hotel and restaurant sector, representing the hospitality industry. Previous work has shown this sector to be particularly subjected to workplace bullying with negative outcomes related to it and has been suggested to be a sector with an aggressive climate [28, 29], making it a suitable population for the investigation of workplace incivility.

## 2. Materials and Methods

### 2.1. Participants

An online survey was completed by 2871 (2058 women and 813 men) members of the Swedish Hotel and Restaurant Workers Union. Participants' ages ranged from 16 to 72 and the mean age was 36.6 years (SD = 12.3). The respondents had been at their current workplace on average for 6.6 years (SD = 7.2), 410 (14%) employees had a managerial or executive position, a majority 2291 (79.8%) were born in Sweden, and 2273 (79%) were in permanent employment. Of the sample, 1188 (41.4%) were service personnel such as waiters/waitresses and receptionists, 1076 (37.5%) kitchen personnel, 45 (12%) facility workers, and 264 (9.2%) belonged to some other category of staff.

### 2.2. Measures

#### 2.2.1. Workplace Incivility

Experienced incivility from supervisor and coworker was measured by the 7-item Workplace Incivility Scale, [19] which was translated into Swedish [30]. The scale assessed the frequency of perceived incivility in the last year, which is a shorter time frame than originally used by Cortina et al. [19].

The scale was modified to measure witnessed workplace incivility, using different stems for the same 7 items, in accordance with Ferguson and Barry's [12] adaptation of the Interpersonal Deviance Scale [31]. Employees were asked to rate how often they have witnessed each of the seven behaviour items in the scale. Example questions were “During the past year while employed in the current organization, have you been in a situation where you have observed any of your superiors: Making demeaning or derogatory remarks about others?” The perception of supervisors and coworkers was rated separately as advocated by Smith et al. [32], for experienced and witnessed incivility.

Consistent with Blau and Andersson [33], the scale was modified to measure instigated workplace incivility. Employees were asked to rate their own behaviour for each of the 7 items in the scale. The response alternatives for all of the incivility measures ranged from 0 (*never*) to 4 (*most of the time*). Cronbach's alphas for experienced incivility from supervisor was .94 and from coworker .92, witnessed incivility from supervisor .96 and coworker .95, and instigated incivility .83.

#### 2.2.2. Organizational Factors

Subscales from the rigorously tested and applied* Copenhagen Psychosocial Questionnaire* (COPSOQ II) [34] in a Swedish variant [35] was used to assess psychosocial factors at work. The subscales were job demands (four items), social support from supervisor (three items), social support from colleagues (three items), and control (four items) having Cronbach's alphas of .80, .90, .80, and .81, respectively. Response alternatives on these scales ranged from 0 (*never/hardly ever*) to 4 (*always*).

#### 2.2.3. Negative Outcomes

Job satisfaction was measured by four items from the COPSOQ subscale. Responses ranged from 1 (*very unsatisfied*) to 4 (*very satisfied*). Cronbach's alpha was .87. Sleeping troubles were captured by four items of perceived sleeping troubles in the last four weeks from the COPSOQ subscale. Responses ranged from 0 (*not at all*) to 4 (*all the time*). Cronbach's alpha was .87. Three items were used to measure turnover intentions among the employees [20]. Responses ranged from 0 (*I strongly disagree*) to 4 (*I strongly agree*). Cronbach's alpha was .79. Well-being was measured by the WHO-Five Well-Being Index [36]. A Swedish version of the instrument was used [37]. The scale consisted of 5 items, ranging from 0 (*never*) to 5 (*all of the time*). Cronbach's alpha was .87.

#### 2.2.4. Demographic Variables

Demographic questions concerned gender, age, supervisor/nonsupervisor, born in Sweden, temporary employment, and length of employment.

### 2.3. Procedure and Ethical Considerations

A link to the online-based survey was presented in a letter directed to the participant with information about the study along with contact information. Participants were free to withdraw at any point. Completing the study was considered consenting to participation.

The survey with the cover letter was forwarded to the Hotel and Restaurant Workers Union, where it was distributed by e-mail through the membership registries. After one week the survey was reissued. As the survey had reached 6800 individuals, roughly 1600 had responded to the questionnaire. As additional reminders went out, around 1200 more members participated, finally resulting in 2871 completed surveys. Ethical approval was granted through the Swedish Central Ethical Review Board.

### 2.4. Strategy of Analysis

To test our hypotheses, we created two structural models, one for hypotheses 1-2 concerning instigated incivility and one for hypotheses 4-5 concerning the negative outcomes of experienced and witnessed incivility in the workplace (it was found that these latent variables correlated −.329, but in the models, when other variables were included, the correlation was insignificant, suggesting that negative outcomes did not add uniquely to instigated incivility when the organizational variables were included, and therefore we decided to make separate models). The incivility variables were estimated as latent variables with the items of each scale as observed variables. Negative outcomes were a latent variable measured by items from the scales job satisfaction, sleeping problems, turnover intentions, and well-being as observed variables. The organizational concepts social support from coworkers and supervisor, control, and job demands were defined as latent variables measured by items from their respective scales.

Since many of the measurement models were based on categorical variables, we estimated them with MPLUS v. 7.11 using the categorical option, estimating with Weighted Least Square with mean and variance adjusted *χ* ^2^ values. This estimation method has been suggested to perform well when variables are categorical. The only exception was the negative outcome measurement model, consisting of summarized scale values and not single items, where we used the Maximum Likelihood Estimator. The fit indices used were CFI values above .95 representing good fit [38] and RMSEA values below .05 representing excellent fit, and values below .07 representing acceptable fit [39], in the measurement models we primarily relied on the CFI since the RMSEA was very unstable and CFI was very close to 1.0, representing almost perfect model fit.

We tested the measurement models of all the latent variables and found that almost all had an excellent fit to the data (CFI > .98), the exception being the model for the negative outcomes. In that model CFI was .92, but after the addition of one error correlation between well-being and job satisfaction the fit was excellent. Loadings for the incivility dimensions were high for all latent variables, in the range between .70 and .95, with a mean loading of .89. With these very good measurement models we were confident that misfit in the structural model could not be attributed to bad measurement models.

**Figure 1:**
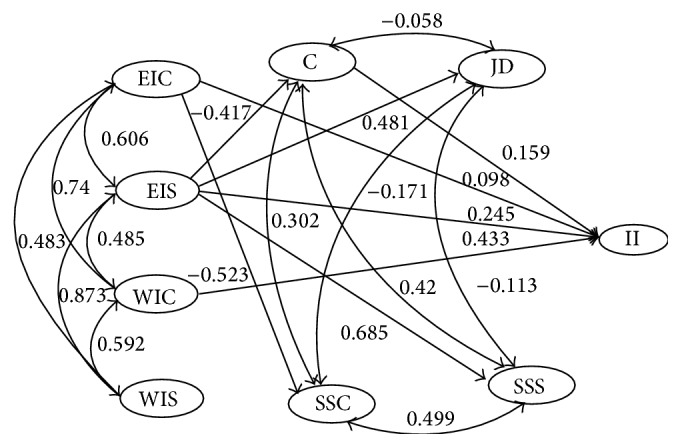
Final structural model showing standardized relationships between the latent variables EIC, experienced incivility from coworker; EIS, experienced incivility from supervisor; WIS, witnessed incivility from coworker; WIS, witnessed incivility from supervisor; C, control; JD, job demands; SSC, social support from coworker; SSS social support from supervisor; and II, instigated incivility. Figures in italics = correlations, figures in plain text = paths (*N* = 2132). All figures are significant at the *p* < .005 level, except the relationship between EIC and II, which was on the border of significance.

The proposed research model for hypotheses 1 and 2 is depicted in Figure 1 and the model for hypothesis 4 and 5 in Figure 3. The only difference is that the dependent variable is instigated incivility in Figure 1 and negative outcomes in Figure 3. We first tested the total fit for each model and after that, based on our hypotheses, we tested for direct effects between the variables in each of the two models as well as mediation of the organizational factors. Since the sample was large the hypotheses were tested with an alpha level of .005.

To test the third hypothesis related to the moderation of the organizational variables in the relationships between incivility (experienced and witnessed) and instigated incivility, a number of latent interaction models were estimated [40]. It was not possible to test all of the interactions in the same model; therefore, the interaction models were simpler in that they consisted of two independent latent interaction variables together with an estimate of their latent variable interaction. Latent interaction variables take a lot of computational resources, making it almost impossible to test more complicated models in MPLUS (to estimate latent interaction variables, mathematical integration is necessary. To make the model less computationally demanding, MPLUS has a procedure that makes this more effective, based on Monte Carlo methods. This method was used in all the presented models including an interaction).

## 3. Results

### 3.1. Descriptive Results

Table 1 displays the correlations, means, and standard deviations for the latent variables in the model.

### 3.2. Instigated Incivility

The first hypothesis concerned whether experienced and witnessed workplace incivility was related to instigated incivility in the workplace. Figure 1 shows the model used for investigating this hypothesis. The full model, including all paths from experienced and witnessed incivility to acting uncivilly, from experienced and witnessed incivility to the organizational variables and from organizational variables to acting uncivilly revealed a very good fit, *χ* ^2^(1090) = 7601.9, RMSEA = .053, and CFI = .974. In this model a number of paths were insignificant (*p* > .10), those paths were set to zero and the model was reestimated. Results revealed an even better fit, *χ* ^2^(1107) = 6614.3, RMSEA = .048, and CFI = .978. In relation to the first hypothesis the most important paths were the ones ending at the instigated incivility latent variable. Three variables were found to have significant paths: the largest was from witnessed incivility from coworkers (*β* = .433, *p* < .001), the second largest from experienced incivility from superior (*β* = .245; *p* < .001). Control (*β* = .159, *p* < .001) also had a significant path. On the border of significance was experienced incivility from coworkers (*β* = .098, *p* = .007). We tested whether witnessed incivility from coworkers had a unique relation to instigated incivility using the MPLUS DIFFTEST. It was found that deleting this path from the model decreased the fit significantly, Δ*χ* ^2^(1) = 144.7, indicating a unique relationship between witnessed coworker incivility and instigated incivility.

Hypothesis 2 concerned mediation effects of the organizational factors. Since perceived control was the only organizational variable that revealed a significant path to the instigated incivility latent variable in the model, and incivility from superior was the only variable with a significant path to perceived control (*β* = −.404, *p* < .001), we only tested mediation effects through this path. It was found that the indirect relationship was significant (standardized specific indirect effect was *β* = −.066; *p* < .001). The total effect (*β* = .179) was slightly lower than the direct path (*β* = .245), suggesting that the direct relationship between control and instigated incivility was suppressed (the indirect and direct effects were also estimated based on MPLUS bootstrap. Using 1000 bootstraps the 99.5% bias corrected CI [−0.099, −0.034] for the standardized indirect effect, and [0.167, 0.323] for the standardized direct effect). To summarize, supervisor incivility was found to predict instigated incivility through perceived low control. In other words, perceived control had an indirect effect on the relationship between experienced supervisor incivility and instigated incivility.

**Figure 2:**
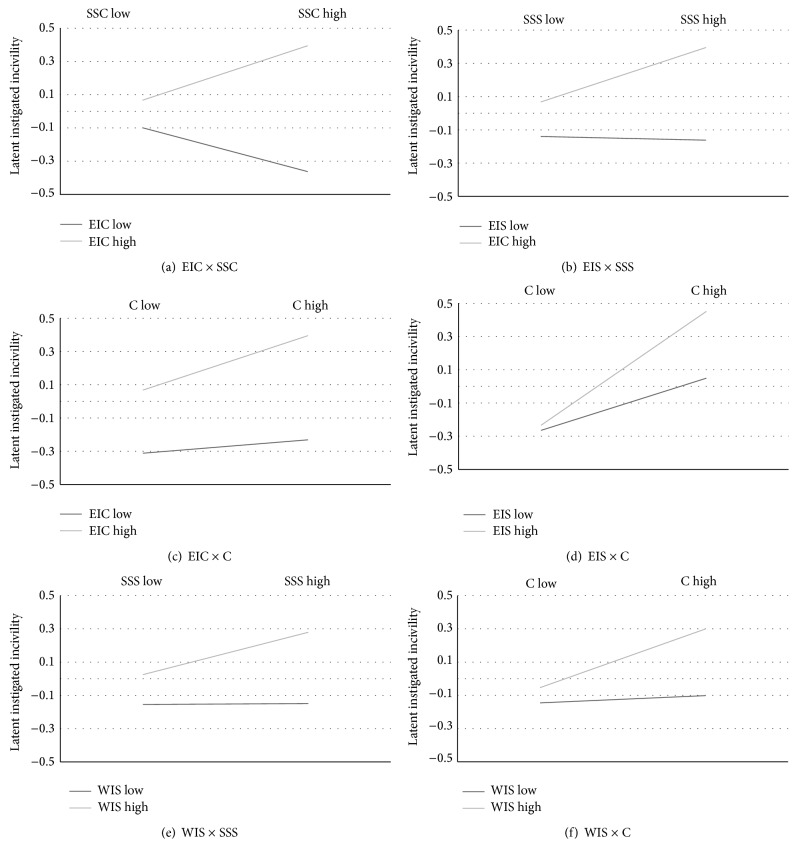
The significant interactions between the latent variables (EIC; experienced incivility from coworker; EIS experienced incivility from supervisor; WIC, witnessed incivility from coworker; WIC, witnessed incivility from coworker; WIS, witnessed incivility from supervisor; SSC, social support from coworker; SSS, social support from supervisor; and C, control) in hypothesis 3 on instigated incivility (*N* = 2132).

Next the moderation models related to hypothesis three were tested. Social support was found to moderate the relationship between experienced and instigated incivility (see the top two panels of Figure 2). Social support from coworkers interacted with experienced incivility from coworkers. Participants high in both these variables tended to report relatively higher instigated incivility (*β* = .148, *p* < .001; coefficients are raw, suggesting that instigated incivility increases by .148 when the product of experienced incivility and social support increases with 1). Social support from supervisor interacted with experienced incivility from superiors. Participants reporting higher levels of incivility from their supervisors together with more support from them also reported more instigated incivility (*β* = .081). Perceived control (see middle panel of Figure 2) moderated both the relationship between experienced incivility from coworkers (*β* = .210, *p* < .001) and instigated incivility, as well as the relationship between experienced incivility from supervisors (*β* = .093, *p* < .001) and instigated incivility. This suggest that subjects who perceive control and at the same time report higher levels of experienced incivility have a tendency to report higher levels of instigated incivility.

In addition, social support from supervisor and control moderated the relationship between witnessed supervisor incivility and instigated incivility (see the bottom two panels of Figure 2). Subjects who had witnessed more incivility from their superiors, who also reported relatively more support from their superiors, tended to report higher levels of instigated incivility (*β* = .062, *p* < .001). Also, subjects who reported having witnessed more incivility from their superiors and experiencing higher level of control reported higher levels of instigated incivility (*β* = .077, *p* < .001). It is important to note that the organizational variables moderate the relationships between incivility and instigated incivility. This suggests that instigated incivility is reported by participants who describe their organization as relatively high in incivility but at the same time perceive that they have support and/or control. Having experienced incivility from superiors and coworkers and witnessed supervisor incivility seems to increase the amount of instigated incivility the participants report when the support or control is perceived as high.

### 3.3. Negative Outcomes

**Figure 3:**
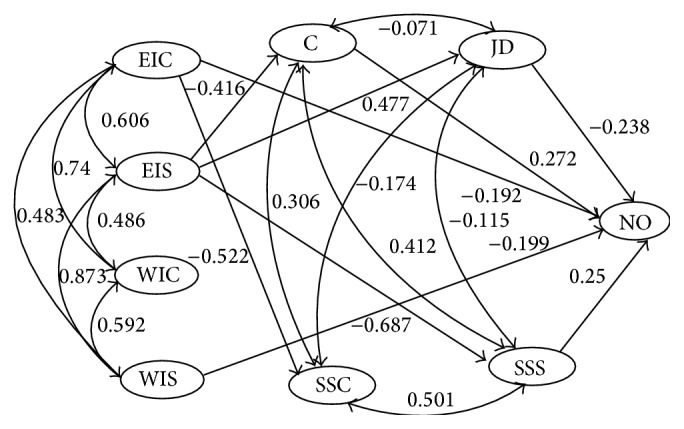
Final structural model showing standardized relationships between the latent variables EIC, experienced incivility from coworker; EIS, experienced incivility from supervisor; WIS, witnessed incivility from coworker; WIS, witnessed incivility from supervisor; C, control; JD, job demands; SSC, social support from coworker; SSS social support from supervisor; and NO, Negative outcomes. Figures in italic = correlations, figures in plain text = paths (*N* = 2132). All figures are significant at the *p* < .005 level.

Figure 3 shows the basic model used when testing hypotheses 4-5 about how experienced and witnessed incivility is related to negative outcomes (well-being, job satisfaction, turnover intentions, and sleeping problems). The full model, including all paths from the four latent incivility variables to the negative outcomes latent variable, with the organizational variables in the middle, had a good fit to the data, *χ* ^2^(953) = 8350.0, RMSEA = .060, and CFI = .966. A model where all paths with standardized coefficients that were insignificant were set to zero had an even better fit, *χ* ^2^(968) = 6639.0, RMSEA = .052, and CFI = .974. In relation to hypothesis 4, two incivility paths were significantly related to negative outcomes, experienced incivility from coworker (*β* = −.192, *p* < .001) and witnessed incivility from superiors (*β* = −.199, *p* < .001). Perceived control (*β* = .272, *p* < .001), job demands (*β* = −.238, *p* < .001), and social support (*β* = .250, *p* < .001) from supervisor also had significant paths to negative outcomes. Experienced incivility from supervisor had significant paths to job demands (*β* = .477, *p* < .001), control (*β* = −.416, *p* < .001), and social support from supervisor (*β* = −.687, *p* < .001). Experienced incivility from coworker had a significant path to social support from coworker (*β* = −.522, *p* < .001), but social support from coworkers did not have a significant path to negative outcomes. Witnessed incivility did not have any significant paths to any of the organizational variables in the model.

Hypothesis 5 concerned the possible mediation of organizational variables on the relationships between incivility and negative outcomes. Possible mediation effects were only tested for experienced incivility from supervisor. This was the only incivility variable correlating with the organizational variables that also correlated with negative outcomes. The total effect from experienced incivility from superiors to negative outcomes was (*β* = −.399, *p* < .001). We first tested a model setting the direct path between experienced incivility from supervisor and negative outcomes to zero. In this model, all indirect effects from experienced incivility from supervisor to negative outcomes were significant; job demands (*β* = −.113, *p* < .001), control (*β* = −.113, *p* < .001), and social support from supervisor (*β* = −.172, *p* < .001). Next, we tested whether estimating the direct path significantly decreased model fit. Testing the difference in *χ* ^2^ with the so called “DIFFTEST” in MPLUS suggested that this addition to the model did not increase fit, Δ*χ* ^2^(1) = 1.001, *p* > .005 (the total indirect effect based on MPLUS bootstrap (1000) had 99.5% CI [−0.467, −0.330], all CI of the single indirect effects excluded zero, and the direct effect was 0.00). To summarize, participants' reported negative outcomes was directly related to their reported experienced incivility from coworker and witnessed incivility from supervisor. Experienced incivility from supervisor had the strongest relation to negative outcomes, but there were no direct effects when the indirect effects through the mediating organizational variables were included in the model.

In relation to the hypothesis 6, the interaction models that were tested for instigated incivility were used with negative outcomes as dependent variable, but none of the 16 possible interactions were found to be significant.

## 4. Discussion

The aim of the study was to examine workplace incivility as a social process, including experienced, witnessed and instigated incivility, and negative outcomes of workplace incivility. The first hypothesis concerning the relationships between experienced and witnessed incivility, and instigating uncivilized acts were partly supported. The study showed a strong and unique relationship between witnessing incivility from coworkers and acting uncivilized, and to some extent being targeted by incivility from a supervisor was also related to instigating incivility. The results are in line with earlier studies by Robinson et al. [41], who found that merely being in a climate of deviance was shown to impact individual deviant behaviour. The present study shows a similar pattern for workplace incivility. In the estimated model witnessed incivility from a supervisor did not have a unique significant relationship to instigated incivility which is in line with the research of Ferguson and Barry [12]. They found that employees adapt to observed behaviours of their colleagues rather than their supervisors. The results expand on the current literature as to include how merely witnessing incivility can impact the individual's behaviour.

The suggestion made by Estes and Wang [4] that incivility should be studied in an organizational context was investigated in the second hypothesis. Perceived lower control mediated the relationship between being targeted by incivility from a supervisor and instigated incivility. As organizational factors may come to impact the perpetration of workplace incivility between employees, incivility should be considered on both an individual and an organizational level.

In relation to the third hypothesis, it was striking that having a socially supportive and controllable environment coupled with high amounts of incivility was connected with more instigated incivility. Literature has previously shown that social support can have buffering effects on workplace bullying [26]. In the present study, however, it was found that high levels of social support from either coworkers or supervisors moderated the relationship between experienced incivility and instigation of more uncivil acts, contrary to a buffering hypothesis. This relationship could possibly be due to a social climate in the organization. Similar aggressive climates in organizations has been discussed by Ramsay et al. [42], where groups with aggressive social rules are more likely to engage in intergroup bullyingand to condone bullying between group members, especially if group members strongly identify with the group. In that way, the socially supportive environment and group cohesion can serve as an enhancement of current group norms in a negative or an aggressive climate. In an aggressive climate, the risk to be excluded or victimized is higher when deviating from the norm [43].

The fourth hypothesis was that experienced and witnessed incivility from supervisor or coworker related to employees' negative outcomes in the form of well-being, job satisfaction, turnover intentions, and sleeping problems. This hypothesis was partly supported, as experienced incivility from coworkers and witnessed incivility from a supervisor were directly related to negative outcomes. The finding that being targeted by incivility from a coworker directly relates to negative outcomes is consistent with previous literature on workplace incivility and detrimental effects on well-being [9, 16], job satisfaction [16, 17], and turnover intentions [6, 19]. It is also consistent with literature on other types of workplace aggression and sleeping problems [21, 22]. The direct relationship of witnessing a supervisor acting uncivilly and negative outcomes is, however, a novel addition to the literature. Turnover intentions have previously been more strongly related to experienced incivility from a supervisor than incivility from a coworker [20]. In the present study, witnessing supervisor incivility had a relationship with negative outcomes, whereas witnessing coworkers acting uncivilly did not significantly relate to negative outcomes. This gives some support to the notion that the relationship found by Leiter and colleagues [20] also can apply in the context of witnessed behaviour.

Employees who are targeted by incivility from a supervisor report more job demands, lower social support and control and as a result they perceive more negative outcomes, as predicted in the fifth hypothesis in the study. The fact that the organizational factors did not mediate the relationship between any other sources of incivility and negative outcomes was not in line with the hypothesis. However, the indirect relationship of experienced supervisor incivility and negative outcomes via organizational factors illustrates the importance of supervisors for the organizational climate and workers' health.

Contrary to hypothesis 6, none of the organizational variables moderated the relationships between any of the incivility variables and negative outcomes. This is not in line with previous studies of the DCS model in organizational research that assessed the buffering effects of demand control and support in relation to well-being [10].

Considering the overall findings, incivility appears linked to a social process in the workplace for both instigated incivility and negative outcomes. Whereas coworker incivility had the largest contribution to explain instigated incivility, experienced supervisor incivility contributed to explain negative outcomes via organizational factors. One should note that witnessed incivility from coworkers interestingly only had a direct path to instigated incivility, and did not make a significant contribution to any of the other tested hypotheses. The findings could be characteristic of the hospitality industry, as it has been pointed out as a sector that could foster aggression [28]. This would explain the counter-intuitive moderation of social support on the relationship between being targeted by incivility and instigated incivility.

### 4.1. Limitations

In relation to our models we report total, direct, mediation, and moderation effects but since the present work is cross-sectional there is no possibility to know if the directions are causal. The high correlations between the latent variables may have revealed one suppressed relationship in the model. This can explain that the total effect (*β* = .179) was slightly lower than the direct path (*β* = .245) between control and instigated incivility.

Moreover, the low response rate could to some extent have limited the study. The low response rate could maybe be due to e-mail administration through the union. As the study was conducted among members of the Hotel and Restaurant Workers Union, largely representing unionized parties of the hospitality industry, the sample is not representative of a general population of the labour market.

The use of the WHO-Five scale in order to measure levels of well-being needs to be considered. More intense testing of the Swedish version of the scale is warranted. However, previous studies have shown that using the WHO-Five rather than other instruments may reduce the risk of ceiling effects [36]. This factor could otherwise risk inducing a false image of severity among the measures. The aforementioned factors may to some extent have limited the study and need to be taken into consideration when interpreting the results.

### 4.2. Future Research

Based on the findings of this study, future research should consider workplace incivility as a social phenomenon. More research is needed concerning the different components of workplace incivility, and their relationships to instigated incivility and negative outcomes. Special attention should be paid to mediating and moderating effects of organizational variables. The results found in the present study support potential indirect paths via organizational variables, but these paths need to be more thoroughly investigated in future research. In addition, testing the moderation effects of organizational factors should be particularly considered in other samples, as the moderating role of a socially supportive environment is a counter-intuitive finding and may even be reversed in other sectors. Longitudinal studies are needed to complement the cross-sectional nature of this studyand address the issue of causality in research on workplace incivility.

## 5. Conclusion

The present research effort shows that workplace incivility was connected to both instigated incivility and negative outcomes in the form of reduced well-being, job satisfaction, turnover intentions, and sleeping problems. Witnessing coworker incivility was the most important dimension to explain instigated incivility. In addition, experienced incivility from coworker and supervisor, as well as witnessed incivility from supervisor, were unexpectedly related to instigated incivility via moderations of perceived high control and high social support.

Negative outcomes were to a high degree explained by experienced supervisor incivility via mediation through perceived low social support, low control, and high job demands. The results emphasize the significance of studying workplace incivility as a social process, considering both experienced and witnessed workplace incivility from coworkers and supervisors in the same model. The results also indicate the importance of including organizational factors as key components in future studies of the research area.

## Figures and Tables

**Table 1:** Descriptives and correlations (ρ) of latent variables included in the models (*N* = 2132).

	1	2	3	4	5	6	7	8	9
Experienced coworker incivility (1)									
Experienced supervisor incivility (2)	.50								
Witnessed coworker incivility (3)	.60	.40							
Witnessed supervisor incivility (4)	.41	.77	.50						
Control (5)	−.21	−.32	−.14	−.26					
Job demands (6)	.24	.33	.22	.31	−.17				
Support coworker (7)	−.41	−.26	−.29	−.20	.26	−.20			
Support supervisor (8)	−.33	−.61	−.28	−.54	.43	−.31	.43		
Negative outcomes (9)	−.40	−.55	−.35	−.51	.41	−.38	.33	.56	
Instigated incivility (10)	.39	.34	.44	.37	−.04	−.17	−.18	−.18	−.23
M (of scales)	6.87	7.71	8.44	8.40	12.72	3.02	6.02	6.33	6.80
SD (of scales)	6.18	7.25	6.69	7.40	5.73	3.27	3.87	3.68	2.83

*Note*. All correlations except the relationship between control and instigated incivility (−.04) were significant at *p* < .001.
